# Exploiting Traffic Light Coordination and Auctions for Intersection and Emergency Vehicle Management in a Smart City Mixed Scenario

**DOI:** 10.3390/s24072036

**Published:** 2024-03-22

**Authors:** Filippo Muzzini, Manuela Montangero

**Affiliations:** Dipartimento Scienze Fisiche, Informatiche e Matematiche, Università di Modena e Reggio Emilia, 41125 Modena, Italy; filippo.muzzini@unimore.it

**Keywords:** smart city, connected vehicles, intersection management, emergency vehicles, vehicle coordination, auction, traffic lights

## Abstract

IoT (Internet-of-Things)-powered devices can be exploited to connect vehicles to smart city infrastructure, allowing vehicles to share their intentions while retrieving contextual information about diverse aspects of urban viability. In this paper, we place ourselves in a transient scenario in which next-generation vehicles that are able to communicate with the surrounding infrastructure coexist with traditional vehicles with limited or absent IoT capabilities. We focus on intersection management, in particular on reusing existing traffic lights empowered by a new management system. We propose an auction-based system in which traffic lights are able to exchange contextual information with vehicles and other nearby traffic lights with the aim of reducing average waiting times at intersections and consequently overall trip times. We use bid propagation to improve standard vehicle trip times while allowing emergency vehicles to free up the way ahead without needing ad hoc system for such vehicle, only an increase in their budget. The proposed system is then tested against two baselines: the classical Fixed Time Control system currently adopted for traffic lights, and an auction strategy that does not exploit traffic light coordination. We performed a large set of experiments using the well known MATSim transport simulator on both a synthetic Manhattan map and on a map we built of an urban area located in Modena, Northern Italy. Our results show that the proposed approach performs better than the classical fixed time control system and the auction strategy that does not exploit coordination among traffic lights.

## 1. Introduction

Smart city scenarios are gaining more and more interest worldwide, as the integration of advanced technologies and intelligent systems is transforming urban landscapes into more sustainable and efficient environments. The advent of the Internet of Things (IoT) [1] and recent advancements in embedded systems have brought significant benefits to the urban viability of Smart Cities. IoT allows large amounts of data about city activities (e.g., traffic, city safety, and current events) to be gathered in real-time; once elaborated, such data become useful information that can be used to improve many different aspects of urban life for citizens and the environment.

We envision a futuristic scenario in which only autonomous and connected vehicles populate the streets and mobility is smart: vehicles will be able to make decisions about their routes by considering many different aspects, such as traffic and weather conditions, the length of the path between the origin and destination, the cost of the route, the driver’s needs, etc. Smart mobility will be possible thanks to the surrounding smart city infrastructure and will result in a reduction of travel times, traffic congestion, pollution, and driver stress while making cities better places to live and work. However, to achieve this scenario many challenges must be addressed, such as the best way to make vehicles go through intersections, how to reduce parking search times, how to facilitate left turns, and more [2].

Among these many issues, in this paper we focus on urban mobility and traffic management, in particular on the crucial challenge posed by intersection management [3]. Indeed, intersections are one of the main causes of delays in city trips today, as multiple traffic flows have to intersect or merge. Therefore, smoothing and facilitating traffic at intersections is a crucial goal in reducing city traffic and the consequent pollution and energy waste.

Traditional traffic lights and precedence rules were originally designed considering a situation where vehicles were solely human-driven and specifically taking into consideration human characteristics and limitations, e.g., different response times, attention levels, and driving skills. On the one hand, traffic lights ensure fairness by allowing all vehicles to eventually pass the crossing by following a First In–First Out (FIFO) policy for each lane. However, the FIFO policy often results in idle vehicles waiting at the intersection even when no other vehicles are approaching from the intersecting lanes. On the other hand, while precedence rules do not suffer from this problem, they may sacrifice fairness. For example, a STOP sign may keep a vehicle idle for a prolonged period if a large number of vehicles with priority continue to arrive. In other words, traffic lights maintain low vehicle latency but do not conserve work, while precedence rules conserve work but can lead to higher latency for certain vehicles.

Intersections are a critical aspect in the management of emergency vehicles; in fact, this is another crucial issue to consider in the design of smart urban solutions [4], as fast response to emergencies can make the difference between saving or not saving lives. Emergency vehicles are used by special services such as police or emergency medical care, and traditionally benefit from special traffic laws that allow them to act differently from other vehicles in order to speed up their arrival at their destination. For example, emergency vehicles are allowed to cross intersections even against a red light. However, such benefits are not always enough. To help emergency vehicles advance faster, it is common practice for other vehicles to try to pull over and make space to let the emergency vehicle pass, even if this is not always possible in heavy traffic conditions and narrow streets and the emergency vehicle has to wait for the lane to get free.

The good news is that, by exploiting real-time data and communication capabilities, smarter and more effective strategies for crossing management can be implemented in the future scenario of a smart city where all vehicles are autonomous and connected: connected vehicles can exchange information both with each other (V2V) and with the infrastructure (V2I), enabling enhanced situational awareness and coordination at intersections [5]. For example, vehicles will not need to come to a complete stop at intersections instead dynamically adjusting their speeds to avoid collisions in crossings [6]. When employing such strategies, the intersection area can be efficiently occupied by more vehicles at a time, resulting in traffic flow optimization and delay minimization for all vehicles involved. This can improve traffic flows, reduce congestion, and improve overall transportation efficiency and security [7], as well as assist in emergency management.

Nevertheless, the complete transition from solely human-driven vehicles to fully autonomous and connected ones will not happen overnight; on the contrary, it is a process that will require a considerable amount of time to reach its culmination. During the initial phase of this transition, human-driven non-connected vehicles will need to coexist with autonomous and/or connected ones, i.e., vehicles possessing communication capabilities will coexist with vehicles without such capability. It is important to observe that the coexistence of connected and non-connected vehicles introduces challenges that do not arise in a scenario with exclusively connected vehicles. Among others, these challenges include: (i) the behavior of non-connected vehicles is unpredictable, and in the worst-case scenario we cannot even expect them to follow traffic rules; (ii) no data can be directly collected from non-connected vehicles, even though some data might be collected by exploiting city infrastructure (e.g., sensors or camera to gather their position); (iii) communication with non-connected vehicles is only possible in limited form and in one direction, i.e., from city infrastructure to vehicle (e.g., traffic lights might still be used to indicate to vehicles/drivers when they are allowed to pass the intersection). Consequently, solutions tailored for scenarios where all vehicles possess communication capabilities may prove ineffective in mixed scenarios. In the latter, additional constraints may need to be added to what vehicles are allowed to do. This mixed coexistence scenario has been much less studied in the literature.

In this paper, we first place ourselves in the mixed scenario, where the distinction between vehicles is determined by their communicative capabilities; then, we propose a novel auction-based intersection management strategy involving (already existing) traffic lights that extends the preliminary proposal appearing in [8] and improves upon the previous proposals in [2,9]. The proposal presented in this paper uses traffic light coordination and bid propagation involving more than one traffic light on a vehicle’s route.

The design of our proposal takes emergency vehicles into consideration as well. When devising a policy for emergency vehicles in a smart city, it is necessary to choose between an ad hoc policy that is activated at need or a general non-ad hoc policy applied to all vehicles (including emergency vehicles) that provides performance guarantees with respect to emergency vehicles. While the former approach allows more freedom in terms of the actions that can be taken for/by emergency vehicles, it must be integrated with the policy designed for other vehicles and requires an ad hoc solution. The latter approach requires more care in the design stage; however, when implemented the system does not need to be aware of whether or not an emergency vehicle is present. The proposal presented in this paper falls into the latter alternative; the system is designed in order to avoid the need for ad hoc solutions for emergency vehicles and to transparently free the routes ahead in order to allow them to arrive quickly at their destination.

We tested our proposal against two baselines: a classical Fixed Time Control system, and an auction strategy without traffic light coordination. Validation was performed through simulations using MATSim, an urban mobility simulator that takes inspiration from the MAS (Multi-Agent System) literature [10]. In MATSim, each road user is modeled as an autonomous agent that features individual and distinct behaviors. Thus, it is possible to model the co-existence of different road users’ categories (i.e., connected and non-connected cars) and examine the results as an emergent global behavior obtained through the interaction among all agents. Simulations were carried out on two different types of map: an artificial Manhattan-style map, and a replica of an area of one square kilometer in the city of Modena, Northern Italy, instrumented with a wide array of smart sensors and communication capabilities in the context of a city project named MASA (Modena Automotive Smart Area). The results show that traffic light coordination is able to reduce traffic times and is a policy suitable for the transition period towards all vehicles being connected. Moreover, emergency vehicles succeed in reducing trip times even in heavy traffic conditions, especially on the map with the largest number of traffic lights.

**Roadmap.** The rest of the paper is organized as follows: in Section 2 we discuss related works; in Section 3 we describe our proposal for traffic light management; in Section 4 we present the experiments we performed to validate our proposal, and report and discuss the obtained results; finally, in Section 5 we conclude the paper with our final considerations and a description of future work.

## 2. Related Work

The recent survey by Mariani et al. in [4] has addressed many aspects and challenges arising in future smart cities. In particular, the authors highlight how coordination among vehicles is a prominent issue in smart city contexts. In the literature, coordination between vehicles is exploited to improve the livability of smart cities by addressing problems such as emergency vehicle management [11] and smart parking management [12,13]. Within their classification of coordination problems for connected autonomous vehicles, in this work we focus on *resource-oriented* issues, more specifically, traffic flow optimization in intersections arbitrated by traffic lights.

The proposals in [14,15,16] are considered milestones in the field, and were among the first to exploit communication between vehicles and city infrastructures to manage intersections in urban areas. The main assumption is that all vehicles are able to communicate either with the smart city infrastructure or among each other, making these methods applicable in the future when all vehicles will be designed to incorporate communication capabilities and/or advanced driving systems. In contrast, in this paper we study the impact of the coexistence of next-generation vehicles with traditional vehicles, and propose approaches that are able to support both types of vehicles. Such a scenario was first addressed in the milestone paper [6], and subsequently in [17]. Here, however, we exploit already existing city infrastructure and take into consideration all vehicles in the lanes, not only the first ones approaching an intersection.

As our proposal addresses traffic light intersection management, we briefly recall some of the main prior results in the following section.

### 2.1. Traffic Lights and Intersection Management

Traditionally, signalized intersections have been managed by static and fixed-timed traffic lights [18]. On the other hand, in [19], which is a foundational paper in vehicle coordination, the authors proposed a method for coordinating both autonomous and human-powered vehicles through traffic lights considering a First Come–First Served policy. A different approach was presented in [20,21], in which two traffic lights competed with each other to reach the highest value of green time. In [2,9], the authors proposed and evaluated how to exploit traffic lights through auction systems for improved traffic management at intersections. Micro-auctions were exploited in [22] in a decentralized manner to determine the next signal phase. Reinforcement learning was adopted in [23] to obtain an optimal bidding strategy. In [24], reinforcement learning was used to learn the traffic signal policy, and other works have used variants of reinforcement learning as well [25,26]. In [27], the authors’ goal was to minimize the personal delay of drivers through an auction for green time. In [28], the authors used a second-price marked approach for auctions. The importance of such auctions is that drivers are able to define their *Value of Time* (VOT), as in [29,30]. Another important aspect is incentive compatibility, as analyzed in [31]. If a driver is not incentivized to adopt the system and to avoid cheating for personal gain against the overall system, then the system performance degrades; this aspect was considered in [32,33]. The authors of [34] focused on CO_2_ emissions. They proposed a smart traffic light system that has the goal of regulating traffic flow to reduce emissions. Multi-intersection road networks were taken into consideration in [35], where the authors proposed a consensus prediction method to coordinate connected vehicles through more intersections. In [36], UAVs were exploited to detect congestion in traffic flows and change traffic light behaviors in response. In [2] and its evaluation in [9] the authors proposed exploiting traffic lights with an auction systems to better manage traffic at intersections. In our work, we extend this concept by enabling coordination among traffic lights in order to obtain a wider view of the actual traffic situation.

### 2.2. Emergency Vehicle Management

Emergency management is one of the crucial issues to be addressed when dealing with traffic flows in smart cities. In [11], the authors proposed a system for clearing the streets traveled by an emergency vehicle communicating with other vehicles. In [37], the authors exploited RFID placement on emergency vehicles to detect them at signalized intersections and grant them a green light. In [38], the authors analyzed the problem of more than one emergency vehicle being present at the same intersection at the same time. In [39], a manual system for changing traffic lights when an emergency vehicle reaches an intersection was proposed based on a mobile application, while in [40] the authors proposed a system based on fuzzy logic that assumes actions can be performed automatically in the smart city. Fuzzy logic was used in [41] as well; the proposed system tries to estimate the arrival time of emergency vehicles at intersections so as to avoid other vehicles being forced to wait. The distributed intersection model [42] was exploited in [43] to help emergency vehicles negotiate with other vehicles to cross intersections. Lastly, in [44] the authors used machine learning to develop a smart pre-emptive traffic signal control system.

In this paper, we propose a system that integrates emergency vehicle management transparently, without the need for ad hoc solutions.

## 3. Proposed Traffic Light Coordination System

In this study, we place ourselves within the context of a smart city scenario in which an urban area is equipped with infrastructure capable of communicating with both central servers and vehicles. We consider a heterogeneous situation as concerns the capabilities of the vehicles roaming the streets; thus, we might have “autonomous” vehicles, i.e., those capable of driving with little or no human intervention, as well as “non-autonomous” vehicles, which are manually driven by human operators. Additionally, we distinguish between “connected” vehicles, those possessing communication abilities with both other vehicles and the city’s infrastructures, and “non-connected” vehicles, which lack such capabilities. It should be noted that connected vehicles are not necessarily autonomous, as non-autonomous vehicles may exploit embedded Internet of Things (IoT) devices or smartphones to communicate with city infrastructure. Within our envisioned scenario, both connected and non-connected vehicles may be autonomous or non-autonomous; these vehicle types coexist, and we concentrate on their different communication capabilities.

To address the challenges posed by this mixed scenario, we propose a traffic light management strategy, called the *Coordinated System with traffic light management* (henceforth the *coordinated system*), with the goal of improving urban mobility. The coordinated system is an auction-based system meant to exploit pre-existing traffic light infrastructure at intersection sites that is able to handle both connected and non-connected vehicles. The coordinated system presented in this paper improves upon the auction-based traffic light management system presented in [2,9] (named the *basic system* from now on) by taking into consideration information gathered by more than one traffic lights and introducing coordination among nearby traffic lights. A preliminary study of this proposal has been presented in [8]. There, coordination occurred only between two consecutive traffic lights; here, coordination is extended to additional traffic lights further along the vehicle’s route. This novelty improves the system’s ability to handle emergency vehicles without the need for any ad hoc policies.

In the rest of this section, we first present the requirements for city infrastructure, then describe how auctions work in the coordinated system.

### 3.1. City Infrastructure

**Street maps.** We assume that there is only one lane in each direction on each street. We represent street maps as graphs, with a node for each crossroad (called *intersections*) and a directed edge (called *links* or *lanes*) for each street connecting two crossroads, directed as the street sense of travel (i.e., if the street is two-way then there will be two directed edges between two nodes). Thus, we refer to outgoing edges as *out-links* and to ingoing edges as *in-links*.

**City server.** The intersection management system has its own dedicated city server that is used to implement coordination among traffic lights. The server can communicate with all traffic lights and has access to the city maps and its IoT infrastructure.

**Traffic lights and intersections.** For intersections with a traffic light, we assume that only one lane at a time displays a green light, while all the other lanes display a red light; all vehicles stop when the light is red and move when the light is green. The length of the green line is fixed and known a priori. The main difference from traditional traffic light management is that only connected vehicles are able to communicate with the traffic light and try to obtain the green light for their lane by means of an auction, as explained in the next section. In order to prevent incidents caused by possibly unpredictable behaviors of non-autonomous vehicles, we do not allow vehicles from different lanes to be in the crossing at the same time, even if they do not have conflicting trajectories. Standard street rules are used to manage intersections without traffic lights.

**Traffic light controller.** We re-use traditional traffic lights that are already present in the city and that have been equipped with a *controller* run on a physical device placed at the intersection site to control the traffic light signal. This device is able to communicate with both the centralized city server and with the connected vehicles approaching the intersection. Furthermore, we assume that the traffic light controllers have access to IoT devices (such as smart cameras, for example) in proximity to intersections that are used to determine the presence of non-connected vehicles and count them.

**Emergency vehicles.** Emergency vehicles are assumed to be connected, as it can be reasonably assumed that in the considered scenario they belong to or are under the control of some city/public institution and have been endorsed with communication power with the aim of improving emergency response and service times.

### 3.2. Traffic Light Auctions

When approaching an intersection controlled by a traffic light, vehicles participate in auctions with the aim of winning the next green light auction for their lane in order to pass through the intersection (or advance in the lane and move closer to the intersection). There is an auction at each traffic light for each green light, where the traffic light controller acts as an auctioneer; it collects all incoming vehicle bids, computes the total lane bids, and decrees the winning lane.

It is not difficult to see that this policy might lead to starvation. For example, starvation might occur for a lane if it has a much smaller number of vehicles with a smaller budget than the other lanes over a prolonged period of time. To avoid lane starvation, the controller keeps track of how far back in time each lane had the last green light; if this was too far back, it automatically adds a huge bid (called a *starvation bid*) to the starving lane to force a green light. At this end of the section, after describing the details of the auction mechanism, we move on to discuss the amount of the starvation bid.

To define the auction strategy, we now describe how bids are computed and placed for both standard and emergency vehicles and how the controller states which lane wins the auction.

**Vehicle budget.** Each connected vehicle is assigned a trip *budget*, which is used to place bids. It is beyond the scope of this paper to define how budgets are assigned to vehicles, as this issue is more an administrative/political one; local administrations might decide to assign budgets according to drivers’ conduct, ecological incentives, average Km/yds traveled per year, etc., or even decide to sell budget.

**Vehicle bids and bidding criteria.** Connected vehicles place bids when approaching intersections controlled by traffic lights. A bid is computed as a vehicle’s total trip budget divided by the number of traffic lights the vehicle will encounter in its route, starting from the current position to the destination. In this way, we ensure that vehicles never run out of budget even if rerouting occurs. Non-connected vehicles are not able to communicate with the controller; thus, in order to take them into consideration, the system places a default bid for each such vehicle. The default bid is calculated as the average of the bids placed by all connected vehicles. As stated before, non-connected vehicles in the proximity of the intersection can be counted by exploiting IoT devices.

**Emergency vehicles.** Exploiting the same idea as in [45], emergency vehicles are assigned a very high budget, but otherwise act as standard connected vehicles. The huge budget is meant to "force" the green light on their lanes and overrule a starvation green light. Following this aim, their budget must be greater than the starvation bid multiplied by the number of traffic lights that the vehicle will encounter along its route.

**Bid propagation.** Vehicle bids in the proximity of a traffic light are used to compute lane bids for the traffic light, and are propagated to the next *p* nearby traffic lights along a vehicle’s route. Propagation scales the bids according to the distance of the further traffic light from the current one. Propagated bids are then taken into consideration to compute those nearby traffic lights’ lane bids. We use bid propagation to improve standard vehicle trip times as well as to allow emergency vehicles a free path by contributing with significant bids to the auction of more distant traffic lights. With this aim, the bid is propagated using a scaling factor equal to $2^{i}$, with i=0,1,…,p−1, i.e., the same bid for the next traffic light is halved at each subsequent one.

In the preliminary version of this paper [8], we showed that propagating the bid to the next traffic light on a vehicle’s route leads to advantages (e.g., trip slowdown reduction) compared to not using propagation. Here, we propose to propagate bids to more distant traffic lights. Preliminary experiments showed that while this extra propagation of halved bids does not affect standard vehicles in an appreciable way, it assists emergency vehicles. This is because the latter have a budget that is orders of magnitude higher than the standard vehicle budget. Thus, extra propagation allows us to maintain the advantages of propagating only to the next traffic light, and provides an advantage to emergency vehicles in a transparent way without the need for an ad hoc policy.

**Figure 1:**
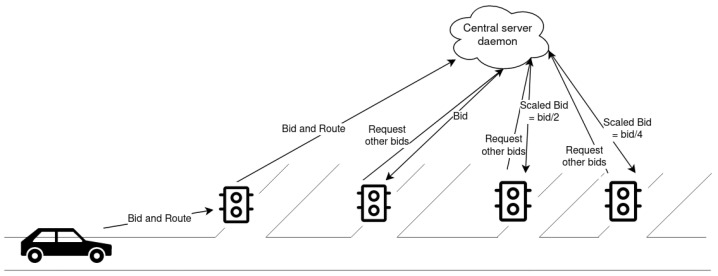
Bid Propagation Flow: the traffic light executes Algorithm 3 to perform an auction, while the central server executes Algorithm 1 when it receives “Bid and route” and Algorithm 2 when it receives “Request other bids”.

In detail, bid propagation works in the following way (see also Figure 1):

- Connected vehicles approaching a traffic light send their bid to the traffic light controller along with the complete route to their destination. The controller collects the information and sends it to the central server.
- For each such vehicle, the server saves the following information: (i) the next (up to) *p* traffic lights on the vehicle’s route; (ii) the current link the vehicle is in; and (iii) the bid placed at the current traffic light (as in Algorithm 1).
- Non-connected vehicles are not able to communicate any of this information to the traffic light controller; therefore, the traffic light controller communicates to the server the number of non-connected vehicles approaching its intersection. For each one, the server makes a random guess about its next lane and next traffic light. This guess is limited to only the next traffic light, as guessing the successive steps can lead to completely wrong conclusions about the vehicles’ routes. The choice is made by selecting the next link that has been selected by the majority of past vehicles.
- When requested by a traffic light, the server then sends the (scaled) bids of the vehicles that are estimated to be at its intersection within a given time lap (as in Algorithm 2). This is estimated using the obtained information about the positions of the vehicles. The time lap is computed as the time needed to reach the traffic light from the vehicle’s current position traveling at the relevant speed limit. For non-connected vehicles, as the route is not known, the bid is propagated only to the next traffic light.

**Algorithm 1** Central server callback when bids are sent by traffic lights **Input** Bids: Vehicles Bids (with the lane and route) NNCs: number of non connected vehicles in the lanes timestamp: time of the call **Variables** TrafficLMap: mapping of traffic lights and bid to propagate statistics: statistics used to sample the next traffic lights for non connected vehicles maxP: max propagation  1:**for** each bid in Bids **do**▹ bids of connected vehicles  2:    p←1  3:    **for** each link in bid.route **do**  4:        **if** link has no traffic lights **then**  5:           continue  6:        **end if**  7:        **if** *p* == maxP **then**  8:           break  9:        **end if**  10:        $propagatedBid.bid\leftarrow bid.amount*0.5^{p}$  11:        propagatedBid.lane←bid.lane  12:        propagatedBid.route←bid.route  13:        propagatedBid.estArrivalTime←timestamp+getTravelTime(lane,link)  14:        TrafficLMap[link].append(propagatedBid)  15:        **if** *p* == 1 **then**  16:           updateStats(statistics,lane,link)  17:        **end if**  18:        p←p+1  19:    **end for**  20:**end for**  21:**for**
each NNC in NNCs **do**  22:    propagatedBid.bid←NNC.deafultBid  23:    propagatedBid.lane←NNC.lane  24:    link←getMostNextTF(statistics,lane)  25:    propagatedBid.estArrivalTime←timestamp+getTravelTime(lane,link)  26:    TrafficLMap[link].append(propagatedBid)  27:**end for**

In order to balance the effect of bid propagation and the communication overload on city infrastructure that it introduces, the value of *p* is chosen as the logarithm of the average of the standard vehicle trip budget. For a given standard vehicle bid *b*, the remaining bid value after logb propagation is negligible, and it is useless to propagate it even further, as this would only negatively engrave communication overload. Moreover, for emergency vehicles it is useless to free the route too far ahead before the arrival of the vehicle.

**Auctions.** In the coordinated system, traffic lights communicate both with other nearby traffic lights and with a dedicated city server to share information about the bids placed by vehicles and to compute their own lane bids. In order to compute the winning lane of the auction (i.e., the one that receives the green light), the traffic light controller proceeds in the following way (see Algorithm 3):

1. The bids coming from vehicles in the lanes (including non connected vehicles default bids) are summed lane-by-lane. The starvation bid is added to lanes that have not had a green light for a long period of time.
2. The controller contacts the central server to retrieve the propagation of the bids placed at previous intersections (if any), then sums them lane-by-lane.
3. The bids are computed lane-by-lane following steps (1) and (2).
4. The green light is set to the lane with the highest total bid.

**Algorithm 2** Central server callback on propagation bid request by traffic lights **Input** lanes: the lanes that the requesting traffic light manages timestamp: time of the call **Variables** TrafficLMap: mapping of traffic lights and bid to propagate thr: time threshold to consider a vehicle incoming  1:**for**
each lane in lanes **do**  2:    **for** each bid in TrafficLMap[lane] **do**  3:        **if** bid.estArrivalTime−timestamp<=thr **then**  4:           result[lane].append(bid)  5:        **end if**  6:    **end for**  7:**end for**  8:return result
**Algorithm 3** Traffic light controller auction algorithm **Input** Bids: Vehicles Bids (with the lane and route) ServerBids: Vehicles Propagated Bids (with the lane) NNCs: number of non connected vehicles in the lanes **Variables** LaneMap: mapping of lanes and cumulative bids LaneVehicles: mapping of lanes and connected vehicles in the lane actualTime: the actual time LastGreenMap: mapping of lanes and the last time in which they had the green light starovationDelta: time that must be elapsed to add the starvation bonus starovationBonus: starvation bonus **Output** The winning lane  1:**for** each bid in Bids **do**▹ bids of connected vehicles  2:    LaneMap[bid.lane]←LaneMap[bid.lane]+bid.amount  3:    LaneVehicles[bid.lane]←LaneVehicles[bid.lane]+1  4:**end for**  5:**for** each lane in LaneMap **do**▹ default bids of non connected vehicles  6:    defaultBid←LaneMap[lane]/LaneVehicles[lane]  7:    LaneMap[lane]←LaneMap[lane]+NNCs[lane]∗defaultBid  8:**end for**  9:**for** each lane in LaneMap **do**▹ starvation  10:    **if** actualTime−LastGreenMap[lane]<starovationDelta **then**  11:        LaneMap[lane]←LaneMap[lane]+starovationBonus  12:    **end if**  13:**end for**  14:**for** each bid in ServerBids **do**▹ propagated bids  15:    LaneMap[bid.lane]←LaneMap[bid.lane]+bid.amount  16:**end for**  17:send Bids and NNCs (with calculated default bid for each lane) to the central server for propagation  18:winner←argmax(LaneMap)  19:LastGreenMap[winner]←actualTime  20:**return** winner

It can be observed that the auction is still performed even if the starvation bid is added to a bid lane. This allows emergency vehicles to “steal” the green light from a starving lane. The starving lane then receives the green light at the next auction.

**Starvation bid.** The starvation bid must ensure that, excepting the presence of an emergency vehicle, a starving lane wins the auction no matter how many vehicles are in the other lanes and how high their bids are. The starvation bid must take into account the number of vehicles approaching the intersection from the other lanes, the bids placed by those vehicles, and possible bid propagation. Thus, it needs to be set to a value larger than the product of the maximum number of vehicles allowed in any lane multiplied by the maximum budget and, to cover for propagation, by the maximum number of lanes at the intersection.

## 4. Experiments and Results

We performed a large set of experiments using the MATSim simulator to test the effectiveness of the coordinated system and evaluate whether it is actually capable of improving urban viability in a mixed scenario where connected and non-connected vehicles coexist. With this aim, we concentrated on evaluating trip times and exploited the simulator’s features concerning time events (e.g., retrieving timestamps for departure and lane entering).

### 4.1. Experimental Setup

We used two different map scenarios: a Manhattan-style artificial map and a real city map. We generated populations composed of vehicles with specific characteristics. In this section, we describe the choices made in our experimental setup.

**Figure 2:**
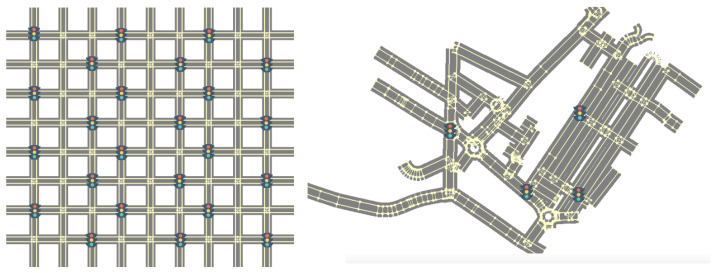
Maps used in our experiments, showing the locations of traffic lights: (**left**) 8 × 9 Manhattan and (**right**) MASA.

*Manhattan scenario.* We used a Manhattan-style map with eight horizontal links intersecting nine vertical links, for a total of 72 four-way intersections, as shown in Figure 2 on the left. Traffic lights were placed randomly and evenly at 24 of these intersections and all four links involved were regulated. Each link contained two lanes, one in each direction. This map did not depict a real city area; rather, it was an artificial map in which each intersection had four links, designed to test the algorithms in a very regular scenario.

*MASA scenario.* The MASA map was a real map of a city area in Modena, Italy called MASA (Modena Automotive Smart Area), shown in Figure 2 on the right. To import the MASA map in MATSim, we used the OpenStreetMap (https://www.openstreetmap.org/ accessed on 15 January 2024) and MATim extensions. We added three traffic lights for our experiments, making for a total of four traffic lights, as the original area has only one traffic light and the experimental results would not have been significant.

MASA is a 1 km wide area equipped with several smart sensors that are able to collect urban and traffic data, and is a smart city testbed involved in the CLASS HORIZON2020 research project (https://class-project.eu/sites/default/files/class_project_files/class_wetice_2019_roberto_presentation.pdf accessed on 15 January 2024) that has been exploited in previous research work to study the behavior of traffic lights [9] and emergency response [11] in smart cities. In this area, connected vehicles can already communicate with a central city server through V2X infrastructure.

*Population.* The population defined the total number of connected and non-connected standard vehicles for the current experiment. Each vehicle was associated with two locations (one standing for the driver’s home and the other one for the driver’s workplace) and with a departure time from home and a return time from work. Home and work locations were randomly generated on the maps to ensure that a path exists from home to work and vice versa (though these paths will eventually be different). Departure and return times were generated according to a normal distribution with peaks at city rush hours (i.e., 09:00 AM for departure and 06:00 PM for return). The vehicle budget was set to a random value uniformly picked in the interval [1,100] in order to account for variability in the vehicles’ available budgets.

*Emergency vehicles.* Emergency vehicles were considered to be connected and to start each trip with a budget equal to the starvation bid multiplied by the maximum number of traffic lights on their route plus one. Routes were generated to ensure that vehicles moved from one side of the map to the other, as short routes are not challenging, and contained at most six traffic lights on the Manhattan map and four on the MASA map.

*Starvation bids and bid propagation.* According to aforementioned constraints, the starvation bid was set to 30,000 and bids were propagated to the next p=5 traffic lights.

**MATSim extensions.** To perform our experiments, we had to implement new ad hoc functions to extend MATSim. Although MATSim has been extensively used in previous research, its baseline version presents several limitations that needed to be addressed; specifically, it does not enable interaction among vehicles and city infrastructure, and it does not contemplate traffic lights. Our implementations were based on previously published MATSim extensions for traffic lights [46] and dynamic agents [47].

### 4.2. Experiments

We tested the coordinated system using both maps while varying the number and type of vehicles in the experimental population. We compared the performance of the coordinated system against three competitors, all working under the same assumptions as our proposal concerning the infrastructure at the intersections while differing only in their intersection management policies:

- The **Fixed Time Controller (FTC) system** simulates the classic round robin-like behavior of traffic light [18]).
- The **Basic System** is an auction-based system with no coordination among traffic lights. The traffic light controller determines the winning lane of the auction using only the bids of incoming vehicles (i.e., it performs only steps (1) and (4) of the coordinated system.)
- The **Coordinated System—Prop** is a nondeterministic version of the proposed coordinated system. Guessing as to the direction of non-connected vehicles is conducted by selecting each outgoing link of an intersection with a probability that is proportional to the number of vehicles that selected the link in the past. This means that the link with heavier traffic has a higher probability of being selected as the next one for non-connected vehicles.

For each experiment we performed twenty runs by varying the population, and we report cumulative statistics on the following metrics computed for each vehicle:

- *Time to Cross*: The time a vehicle spends during its route waiting in line to move through an intersection regulated by a traffic light. This is influenced by traffic and by the number of red lights encountered during the route, with the smallest number being the best. We report this measure for both the Manhattan and MASA maps. We highlight the outliers in the reported plots, as they are the most interesting part.
- *Green Traffic Lights Percentage*: The percentage of green traffic lights (i.e., no waiting) with respect to the total number of traffic lights encountered along the route. In this case, the largest number is the best. We report this measure for both the Manhattan and MASA maps. For readability, our plots do not report results for the FTC system, as this percentage is always much smaller than the same value for the other systems and in a large majority of cases it is zero.
- *Trip Time Slowdown*: The total time traveled by a vehicle from departure to destination divided by the time needed to travel the same route without traffic and with all green lights. This is a measure of how much the introduction of traffic lights and the presence of traffic affects the time to destination. It is directly influenced by traffic and the number of encountered traffic lights, and indirectly by the length of the route (the longer the higher the route, the higher the probability of spending time in traffic). The value is always at least one, and the smallest number is the best. We report this measure for both the Manhattan and MASA maps.

The first two metrics allow us to understand what happens to vehicles during their trips, and the third to determine whether the system is able to regulate traffic such that vehicles reduce their trip times and arrive at their destination earlier.

In summary, for each experiment we report six plots: (a) time to cross in the Manhattan map; (b) time to cross in the MASA map; (c) green traffic lights percentage on the Manhattan map; (d) green traffic lights percentage on the MASA map; (e) trip time slowdown on the Manhattan map; and (f) trip time slowdown on the MASA map. Plots (a), (b), (e), and (f) are in logarithmic scale, while (c) and (d) are not.

**Experiment 1 (all connected).** In this experiment, the population is composed entirely of connected vehicles. We vary the number of vehicles to understand how the system performs under different traffic conditions in what is supposed to be the most ideal scenario, i.e., when all vehicles are able to place their own bid to participate in the auction.

We progressively increased the number of vehicles starting from 500 (very light traffic) up to 5000 (very heavy traffic) in steps of 500 in order to investigate different traffic conditions.

**Figure 3:**
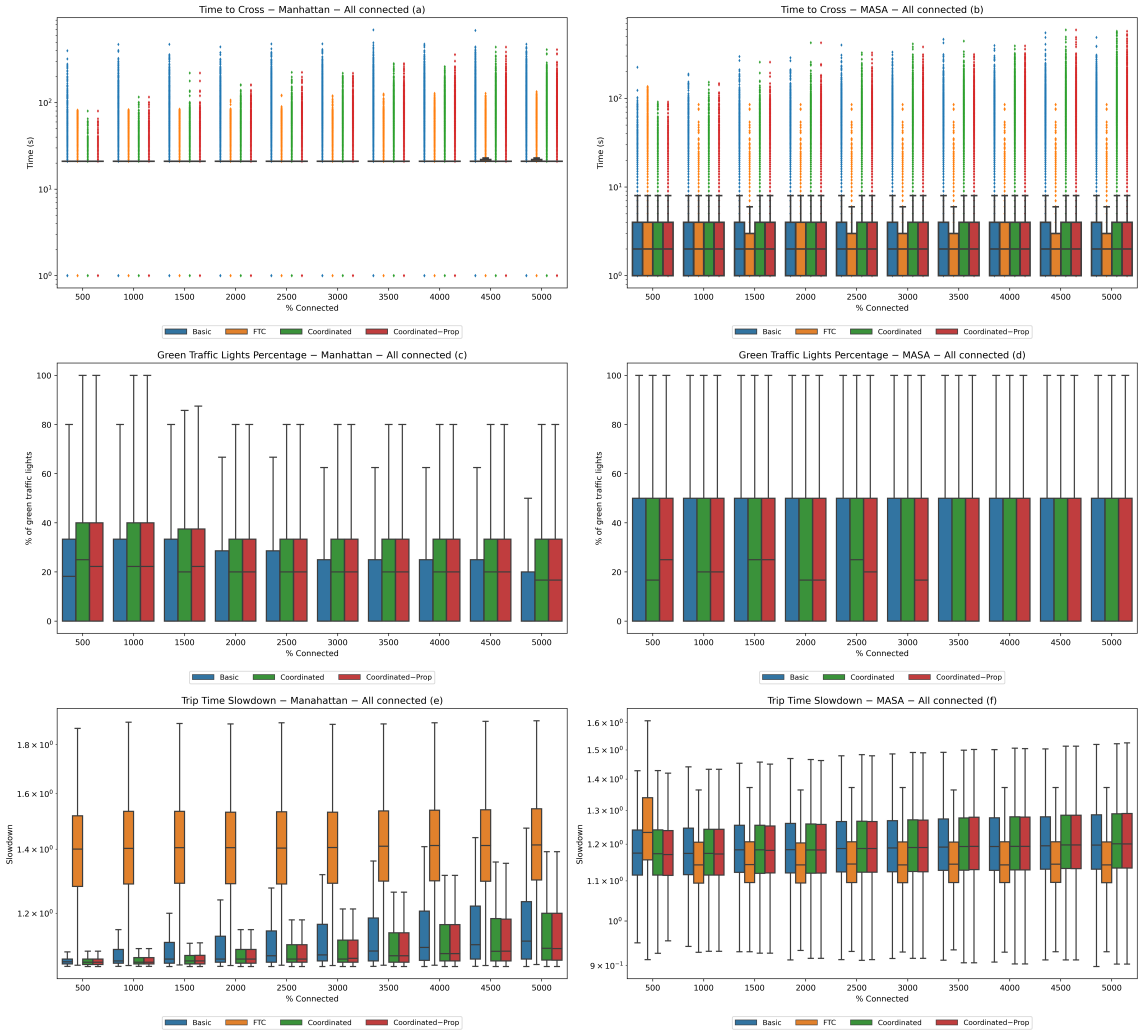
**Experiment 1.** All connected vehicles, varying traffic conditions. Statistics for the Manhattan (**a**,**c**,**e**) and MASA (**b**,**d**,**f**) maps.

The results are shown in Figure 3. In general, it can be seen that the performance grows worse as traffic becomes heavier; this is more evident on the Manhattan map than on the MASA map, as the latter has a smaller number of traffic lights. It can additionally be observed that there is no appreciable difference between the deterministic and nondeterministic versions of the coordinated system.

Concerning the time to cross, the difference among the policies is shown by the outliers, reflecting the different rationales behind the fairer FTC system and less fair auction-based systems. The boxes are flattened on the medians, while the medians are all the same; however, the FTC outliers (even with heavy traffic) are always smaller than for the other policies. Nonetheless, the results show that in the coordinated systems the outliers take less time to cross than with the basic system with no coordination or propagation.

For the Manhattan map, the FTC outliers are at least one quarter less than the worst performing basic system, and at are least half as much as the coordinated system. The coordinated systems with light traffic show outliers that are closer to those of the FTC, while for average and heavy traffic the outliers are about double those of the FTC ones and about two-thirds those of the basic system. For the MASA map, the differences between the basic system and the coordinated ones are smaller, the main reason being the smaller number of traffic lights.

As concerns the percentage of green lights, it can be seen that on the Manhattan map the coordinated systems outperform the basic system, while on the MASA map the results are flattened again due to the smaller number of traffic lights.

As concerns the trip slowdown, it is apparent that the results are different for the two maps. On the Manhattan map, the FTC policy is outperformed by the auction-based policies and the coordinated ones outperform the basic system. It can be seen that all systems perform better with lighter traffic than with average or heavy traffic. On the MASA map, with the exception of very light traffic conditions, FTC always outperforms the other systems.

**Experiment 2 (coexistence).** In this experiment, we progressively increased the ratio of non-connected vehicles over a fixed population number in order to better understand the robustness of the system against the presence of a percentage of vehicles that do not participate in the auction with their own bid (i.e., with only the default one) and with shorted propagation.

**Figure 4:**
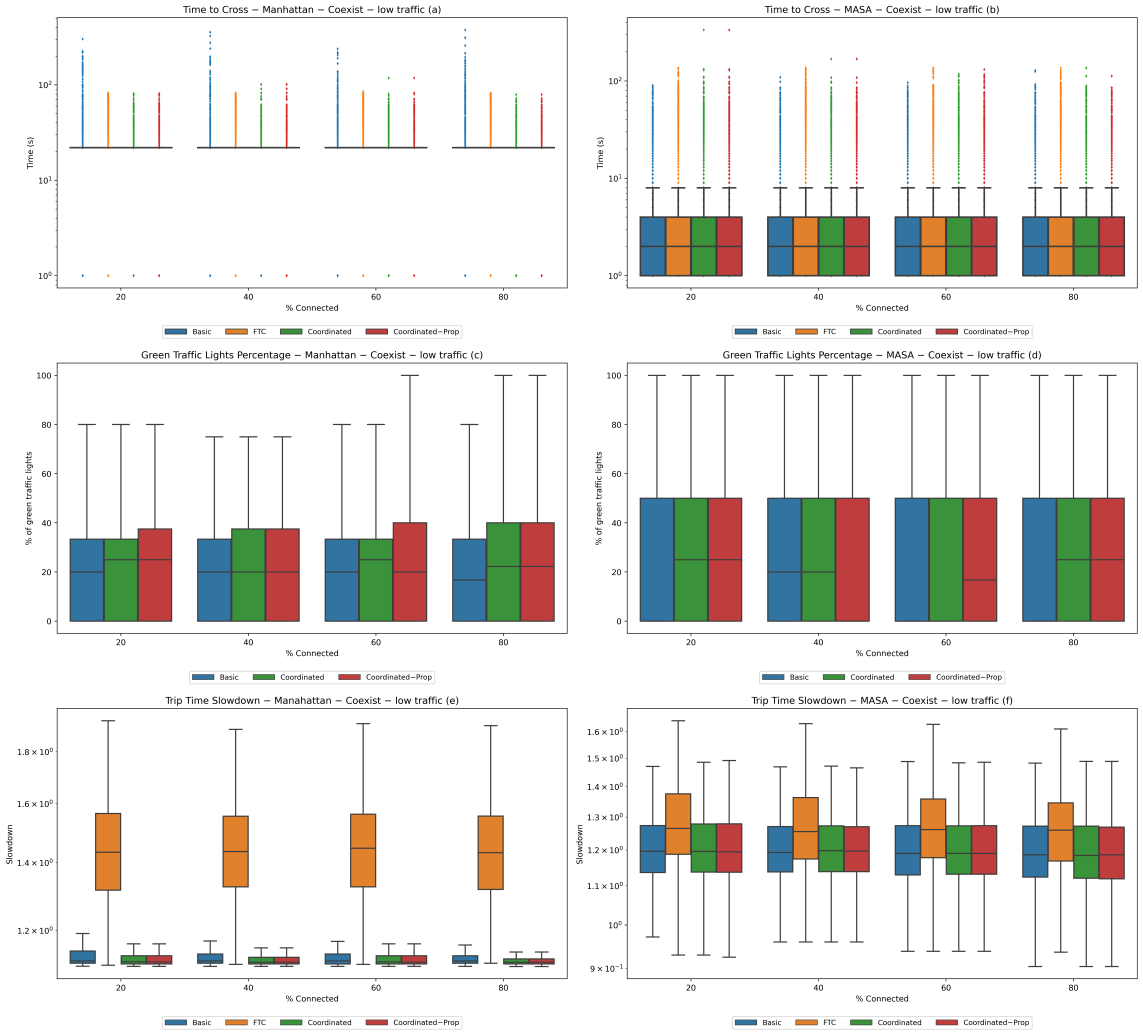
**Experiment 2—low traffic.** Coexistence of connected and non-connected vehicles with 1000 total vehicles. Statistics for the Manhattan (**a**,**c**,**e**) and MASA (**b**,**d**,**f**) maps.

**Figure 5:**
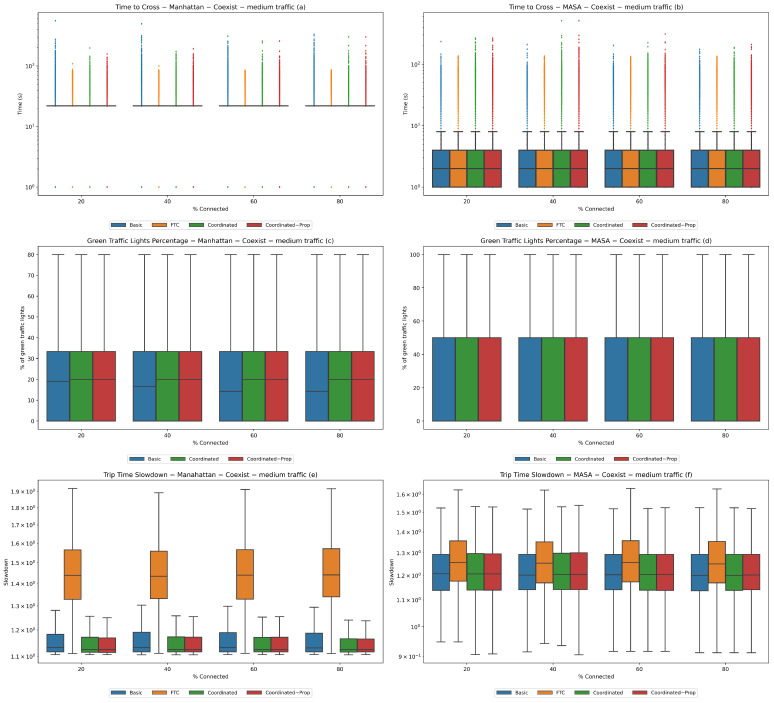
**Experiment 2—medium traffic.** Coexistence of connected and non-connected vehicles with 2500 total vehicles. Statistics for the Manhattan (**a**,**c**,**e**) and MASA (**b**,**d**,**f**) maps.

**Figure 6:**
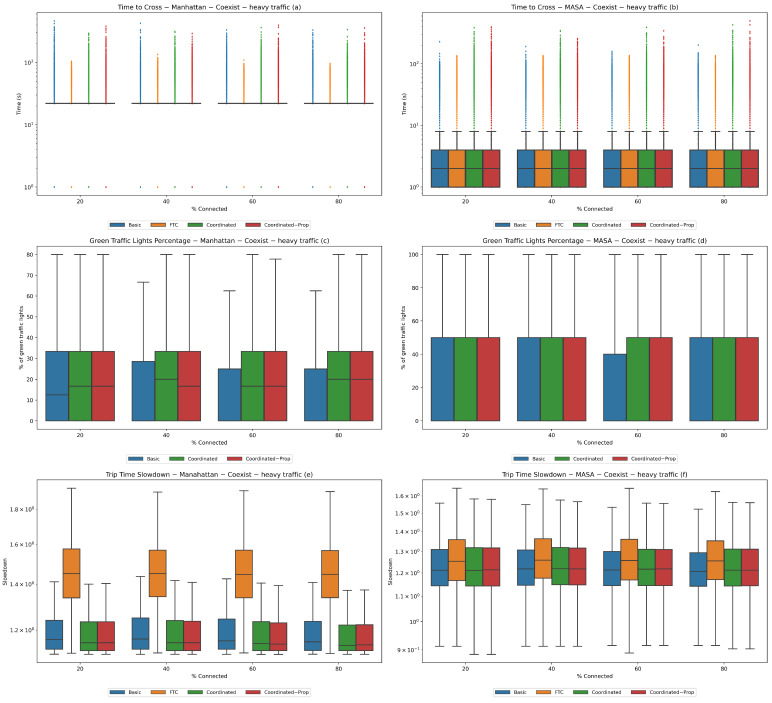
**Experiment 2—heavy traffic.** Coexistence of connected and non-connected vehicles with 4000 total vehicles. Statistics for the Manhattan (**a**,**c**,**e**) and MASA (**b**,**d**,**f**) maps.

We performed this experiment in three different traffic conditions: low traffic with 1000 vehicles, average traffic with 2500 vehicles, and heavy traffic with 4000 vehicles. We started with 20% connected vehicles and increased the percentage by 20% in each successive population up to 80%. The results are reported in Figure 4, Figure 5, and Figure 6, respectively.

It can be seen that there are several common trends across the experiments: (i) the coordinate systems are always comparable, often behaving in the same way and outperforming both the basic system and FTC; (ii) performance improves as more connected vehicles are introduced to the Manhattan map, and is practically stable on the MASA map; and (iii) performance worsens with increasing traffic.

Trends (ii) and (iii) are to be expected, as fewer connected vehicles and more traffic are worst-case scenarios. It is interesting, however, to notice that the performances concerning trip time slowdown is always better on the Manhattan map than on the MASA map. Indeed, slowdown values are scattered within a much smaller interval for the Manhattan map, and the interval is included in the one relative to the MASA map, where medians are always higher. This happens even if the Manhattan map has a larger number of traffic lights than the MASA map, suggesting that the way in which traffic lights manage intersections has a positive impact on traffic flows on the whole map.

**Experiment 3 (emergency vehicles).** In this experiment, we tested whether the coordinated system is able to privilege emergency vehicles only by assigning them a very high budget, and compared its performances with the basic and FTC systems as well as with the coordinated system when the bid is propagated without scaling to the next traffic light only (i.e., the proposal in the preliminary version of this paper [8]). The latter experiment used two versions depending on the guess of the next lane for non-connected vehicles. Analogous to the current proposal, *Coordinated—NO Propagation* selects the most traveled next lane and *Coordinated—Prop—NO Propagation* selects the next lane randomly according to a probability that reflects how many vehicles have chosen each lane in the past.

**Figure 7:**
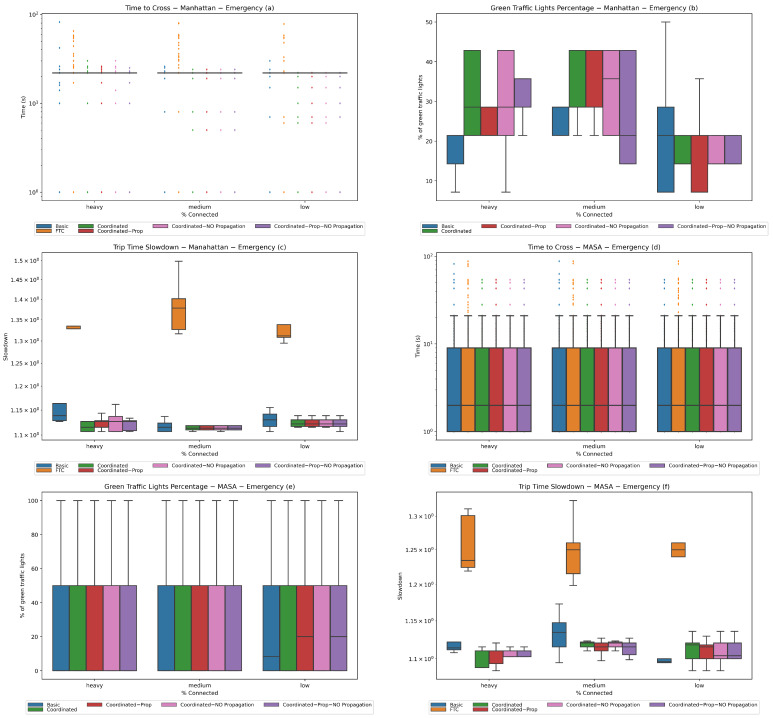
**Experiment 3.** One emergency vehicle in low, average, and heavy traffic conditions (1000, 2500, and 4000 vehicles, respectively) and 60% connected vehicles. Statistics for the Manhattan (**a**,**c**,**e**) and MASA (**b**,**d**,**f**) maps.

In this case, experiments were also conducted on the three different traffic scenarios with 1000, 2500, and 4000 vehicles (low, average, and heavy traffic, respectively) and with exactly one emergency vehicle. The latter used a budget equal to 210,000, which is sufficiently large according to the discussion in Section 3.2. The population for each experiment used 60% connected vehicles. The results are shown in Figure 7 and refer to the emergency vehicle only.

As concerns the time to cross, it can be observed here that the outliers of the FTC system are the worst, followed by the basic system and then by the coordinated systems. The latter show no appreciable differences. Here, the fairness of the FTC system plays against emergency vehicles.

For the percentage of green traffic lights, again there are no appreciable differences among the coordinated systems on the MASA map, while the situation is more interesting on the Manhattan map. Here, it can be seen that the basic system is competitive only in low-traffic situations, while the coordinated system is comparable to the coordinated systems without propagation. With medium traffic, the coordinated systems with propagation are comparable to or outperform the ones without propagation. Finally, in heavy traffic the coordinated system with propagation outperforms the coordinated system with propagation, and is a good competitor for the coordinated systems without propagation as well. In fact, it can be seen that the coordinated system without propagation has the same box but has a whisker indicating smaller values, and that the coordinated-prop box and its whisker are included in the coordinated system box.

For trip slowdown, we observe that the coordinated system has the best performance on both maps with heavy traffic; slowdown values are comparable to those occurring for the vehicles in the low traffic scenario, with smaller variances. This means that the emergency vehicle’s trip time is faster than that of the other vehicles in the best scenario, indicating that the proposed strategy works. It can additionally be seen that the FTC system has the worst performance on both maps. On the Manhattan map, the basic system has the worst performance among the auction-based systems, especially with heavy traffic, while on the MASA map it is competitive only with low traffic.

### 4.3. Discussion

The findings of our experiments suggest that urban mobility could see improvements through the implementation of the proposed coordinated system. This is especially notable in situations featuring numerous traffic lights and larger percentages of connected vehicles when compared to both the non-coordinated auction system and the conventional FTC system. Moreover, the proposed system shows potential in handling emergency vehicles.

The trip time slowdown results are better for the Manhattan map as compared to the MASA map, suggesting that the system is more suitable for maps with a larger number of traffic lights. Indeed, on the MASA map (or maps with a small number of traffic lights in general), factors beyond coordinated auctions at traffic lights play a more significant role in trip time determination. This outcome can be explained by observing that minimal coordination alone cannot address traffic flow challenges, affirming the effectiveness of the coordinated system in reducing traffic congestion when coordination is sufficiently substantial.

It notable that even if the time to cross is always smaller in the FTC system, this system has always the worst slowdown. While this might seem contradictory, it can be explained by further analyzing the policies and results. FTC is a fair policy that is able to lower waiting times at a single traffic light; however, it is not able to reduce the number of red lights encountered in the same way as the coordinated system does. In the latter system, while vehicles might wait for a longer (and less predictable) time at each traffic light, the system is able to decrease the number of red lights encountered during the trip, resulting in a shorter overall trip duration.

As already mentioned, we observed a general enhancement in performance when increasing the percentage of connected vehicles and a decline when the overall number of vehicles increases. These two phenomena were expected, as more connected vehicles makes the traffic light coordination more effective and heavier traffic makes any policy less effective. The first result is a positive one for the transitional period, as it means that the proposed system can be used throughout this period with increasing benefits for the whole population.

It is interesting to notice, however, that the guessing strategy for forecasting the next link of non-connected vehicles does not seem to be determinant. The performances of the two strategies (proportional and deterministic) do not show sensible differences except for the emergency vehicle. These results suggest that non-connected vehicles traveling on main streets are a good approximation of their actual distribution when considering the whole scenario. Moreover, with fewer non-connected vehicles the impact of incorrect guesses for the next lane is smaller the. On the other hand, concentrating the bids of non-connected vehicles to the more crowded lanes seems to help emergency vehicles clear these crowded lanes.

Finally, when dealing with emergency vehicles, traffic light coordination and bid propagation show better results than no coordination and no propagation as concerns the trip time slowdown, especially in heavy traffic conditions. This result is interesting because the adopted policy does not require any special treatment for emergency vehicles, with this instead being naturally integrated into the proposed intersection management strategy.

## 5. Conclusions and Future Work

In this work, we have focused on signalized intersection management using auction-based systems, in particular the scenario in which connected and non-connected vehicles coexist. We have proposed an auction-based system to manage intersections by exploiting coordination of traffic lights along vehicles’ routes is to manage emergency vehicles without introducing ad hoc policies.

Our proposal was tested through extensive simulations. The results are promising, indicating that the coordinated system holds its own against non-coordinated auction-based systems and surpasses the FTC system for emergency vehicle management. Furthermore, the performance of the proposed coordinated system improves with a higher percentage of connected vehicles, making it suitable for the transitional phase while providing incentives for the transition to connected vehicles.

Future works include deeper analysis of some of the presented results to understand whether this proposal can be improved and to determine any intrinsic limitations. For example, we wish to study the scaling bid propagation factor and compare the performance of emergency vehicles to other systems in which ad hoc policies are implemented. Moreover, we intend to investigate whether increasing the number of traffic lights that are coordinated might bring about further improvements to traffic flows and times. An additional area of investigation involves different bidding strategies and their comparison to the one that has been implemented in this paper. We wish to understand how the way in which bids are computed affects the system’s overall performance.

Considering intersection management from a wider perspective, we intend to investigate how other aspects of smart cities and urban transportation might be exploited and incorporated, along with other vehicle characteristics such as electric vehicles or innovative infrastructure alternatives.

## Data Availability

Data are contained within the article.
